# Preoperative intravenous iron treatment reduces postoperative complications and postoperative anemia in preoperatively anemic patients with colon carcinoma

**DOI:** 10.1007/s00384-021-04080-9

**Published:** 2021-12-20

**Authors:** Mikael Kangaspunta, Jorma Mäkijärvi, Selja Koskensalo, Arto Kokkola, Perttu Arkkila, Tom Scheinin, Suvi Rasilainen

**Affiliations:** 1 Faculty of Medicine, University of Helsinki, Helsinki, Finland; 2 Department of GI Surgery, Abdominal Centre, Helsinki University Hospital and University of Helsinki, Helsinki, Finland; 3 Department of Gastroenterology, Abdominal Centre, Helsinki University Hospital, University of Helsinki, Helsinki, Finland

**Keywords:** Anemia, Intravenous iron, Colon cancer, Colorectal surgery

## Abstract

**Purpose:**

Anemia is common among patients with colorectal cancer and is associated with an increased risk of complications and poorer survival rate. The main objective of our study was to determine the effect of preoperative intravenous iron supplementation therapy on the need for red blood cell transfusions, other postoperative complications, and length of hospital stay in colon cancer patients undergoing colon resection.

**Methods:**

In this retrospective cohort study, data were collected from medical records of all 549 colon carcinoma patients who underwent a colon resection in Helsinki University Hospital during the years 2017 and 2018. The patients were divided into two cohorts: one with anemic patients treated with preoperative intravenous iron supplementation therapy (180 patients) and one with anemic patients without preoperative intravenous iron supplementation therapy (138 patients). Non-anemic patients and patients requiring emergency surgery were excluded (231 patients).

**Results:**

Patients treated with intravenous iron had less postoperative complications (33.9% vs. 45.9%, *p* = 0.045) and a lower prevalence of anemia at 1 month after surgery (38.7% vs. 65.3%, *p* < 0.01) when compared with patients without preoperative iv iron treatment. No difference was found in the amount of red blood cell transfusions, length of stay, or mortality between the groups.

**Conclusion:**

This is the first study demonstrating a significant decrease in postoperative complications in anemic colon cancer patients receiving preoperative intravenous iron supplementation therapy. This treatment also diminishes the rate of postoperative anemia, which is often associated with a facilitated recovery.

## Introduction

*Anemia* is defined by the World Health Organization as having blood hemoglobin levels of less than 130 g/l in men and less than 120 g /l in non-pregnant women. Iron deficiency is common in patients with colorectal cancer (being present in up to 48% of patients) and 66% of them are anemic [1]. Of all patients with colorectal cancer who were undergoing surgery, up to 56% are anemic [2]. Anemia has been associated with increased risk for perioperative complications and a poorer survival rate [3, 4]. Recently, iron deficiency has also been linked to increased oncogenicity in cancer patient due to irons vital role in the maintenance of immunological functions [5]. In addition, allogeneic red blood cell transfusions (RBCTs), which are often used to treat anemia, have been associated with an increased complication rate, including increased mortality and a worse oncological outcome [6, 7].

Intravenous iron supplementation has been studied as a treatment method for iron-deficiency anemia among patients with colorectal cancer who are undergoing surgery. It has been described to be effective at treating preoperative anemia [8] and more effective than oral iron at preventing postoperative anemia [9]. It has also been associated with significantly lower amounts of allogeneic RBCT and a shorter length of stay (LOS). A trend towards a lower complication rate has been reported [10]. Some studies show conflicting evidence about the effect of iv iron on RBCT [2, 9–13]. Evidence on how iv iron affects long-term survival in colon cancer patients is still unclear. According to a current consensus statement, iv iron “should be used as front-line therapy in patients who do not respond to oral iron or are not able to tolerate it, or if surgery is planned for < 6 weeks after the diagnosis of iron deficiency” [14].

The main objective of this study was to determine the effect of preoperative iv iron in colon cancer patients undergoing surgery. The primary outcome was the difference in the number of patients receiving RBCT (up to 30 days after the operation) between the two study groups. Secondary outcomes included differences in the following: the prevalence of postoperative anemia at 1 month after surgery, the number of surgery-related complications (up to 30 days after surgery), 30- and 90-day mortality, and length of hospital stay. We hypothesized that preoperative iv iron treatment would decrease the demand for RBCT and diminish the rate of postoperative anemia by 30% and postoperative complications by 15% in anemic patients with colon carcinoma.

## Materials and methods

### Patient population

In this retrospective study, data was collected from the medical records of colon cancer patients operated on at Helsinki University Hospital (Jorvi) during the years 2017–2018. From 2017 onwards, the use of preoperative iv iron has increased in our clinic due to the increased knowledge on the perioperative problems caused by anemia. All consecutive patients were included, numbering 549 in total. The patients were split into two cohorts: one with anemic patients treated with preoperative iv iron up to 60 days prior to the operation (180 patients) and the other with anemic patients without iv iron treatment within the last 60 days prior to the operation (138 patients). Patients with emergency surgery (45 patients), non-anemic patients (160 patients), and patients with iv iron without anemia (26 patients) were excluded from the statistical analysis. Patients who had received iv iron over 60 days prior to the operation were included in the control group (21 patients). The World Health Organization’s definition of *anemia* was used as a selection criterion in this study. The study protocol was approved by the institutional review board of Helsinki University Hospital (Approval ID HUS/333/2019).

### Intravenous iron

Patients in the iv iron group received either 500 mg or 1000 mg IV ferric carboxymaltose at a time, according to general instructions and depending on the patient’s weight and hemoglobin level. The infusion was mostly carried out at an out-patient clinic. Forty patients received 500 mg, 1 patient received 800 mg, 109 patients received 1000 mg, 1 patient received 500 mg + 500 mg, 9 patients received 1000 mg + 500 mg, 9 patients received 1000 mg + 1000 mg of IV iron, and 1 patient received 500 mg + 1000 mg + 1000 mg. For 10 patients, the exact information about the dosage was not available.

### Surgery and enhanced recovery after surgery

All patients were treated within an institutional enhanced recovery after surgery (ERAS) program that included all the important items: preoperative information and optimization, the optimized preoperative glucose balance, standardized anesthesia protocol, mini-invasive surgery (whenever possible), opioid-sparing pain management, prompt postoperative mobilization, and early per-oral nutrition.

The majority of patients were operated on by the standard laparoscopic methodology with 12 mmHg intra-abdominal pressure (IAP). The site of bowel-specimed extraction was shielded with a wound protector (Alexis® AppliedMedical). In the case of a laparotomy, the wound was uniformly protected. No standard postoperative drainage or nasogastric tube was used.

### Outcomes

The primary outcome of this study was to demonstrate the difference in the number of patients receiving RBCT (up to 30 days after the operation) between the two study groups.

The secondary outcomes included the prevalence of postoperative anemia at one month after surgery, the amount of surgery-related complications other than RBCT (up to 30 days after surgery), 30- and 90-day mortality, and length of hospital stay. All complications were recorded using an upgraded Clavien-Dindo classification for colorectal surgery [15].

### Statistical analyses

The statistical analysis was carried out using IBM SPSS statistics 25 software. A chi-squared test was used to assess the difference between cohorts in RBCT, surgery-related complications, the prevalence of anemia, and 30- and 90-day mortality. A Mann–Whitney *U*-test was used to assess the LOS and number of RBC units. Frequencies and descriptives were used to analyze the characteristics of the patients. A 95% confidence interval was used in the analysis. A *p* value < 0.05 was considered statistically significant.

## Results

### The characteristics of the patients

Detailed characteristics of the patients are presented in Table 1. The patients in iv iron group and control group did not significantly differ in their basic characteristics. Median age and BMI were similar, median ASA classification (American Association of Anestesiologists), and Charlsons comorbidity index were equal.

**Table 1:** The characteristics of the patients

	iv iron	Control
Number of patients	180	138
Gender		
Male (%)	84 (46.7)	72 (52.2)
Female (%)	96 (53.3)	66 (47.8)
Age (median [IQR]), years	73.8 (66.9–80.1)	76.0 (70.1–81.9)
BMI (median [IQR]), kg/m^2^	25.4 (23.1–29.1)	25.8 (23.4–30.3)
Location of the tumor		
Sigmoid colon (%)	28 (15.6)	39 (28.3)
Descending colon (%)	2 (1.1)	5 (3.6)
Splenic flexure (%)	17 (9.4)	2 (1.4)
Transverse colon (%)	12 (6.7)	18 (13.0)
Hepatic flexure (%)	23 (12.8)	6 (4.3)
Ascending colon (%)	47 (26.2)	39 (28.3)
Caecum (%)	51 (28.4)	29 (21.0)
Grade of the tumor		
1 (%)	13 (7.2)	13 (9.4)
2 (%)	138 (76.7)	103 (74.6)
3 (%)	20 (11.1)	14 (10.1)
4 (%)	0 (0)	1 (0.7)
Unknown (%)	9 (5.0)	7 (5.1)
Stage		
1 (%)	21 (11.7)	23 (16.7)
2 (%)	82 (45.6)	58 (42.0)
3 (%)	60 (33.3)	41 (29.7)
4 (%)	17 (9.4)	16 (11.6)
Type of the procedure		
Laparoscopic (%)	130 (72.2)	95 (68.8)
Laparotomy (%)	50 (27.8)	43 (31.2)
Indication		
Curative (%)	168 (93.3)	124 (89.9)
Palliative (%)	12 (6.7)	14 (10.1)
ASA classification		
1 (%)	4 (2.2)	2 (1.4)
2 (%)	63 (35.0)	32 (23.2)
3 (%)	83 (46.1)	76 (55.1)
4 (%)	30 (16.7)	28 (20.3)
5 (%)	0 (0)	0 (0)
Median (IQR)	3 (2–3)	3 (2.75–3)
Charlson Comorbidity Index		
Median (IQR)	6 (5–7)	6 (5–8)
Adjuvant chemotherapy		
Yes (%)	78 (43.4)	50 (36.2)
No (%)	102 (56.7)	88 (63.8)

Outcome variables are presented in Table 2.

**Table 2:** Outcome variables

	iv iron	Control	*p*
Red blood cells			
Preoperatively (%)	50 (27.8)	28 (20.3)	0.12
Intraoperatively (%)	28 (15.6)	29 (21)	0.21
Postoperatively (%)	21 (11.7)	23 (16.7)	0.20
Red blood cell units			
Preoperatively (mean ± SD)	0.82 ± 1.6	0.57 ± 1.5	0.09
Intraoperatively (mean ± SD)	0.25 ± 0.69	0.28 ± 0.60	0.24
Postoperatively (mean ± SD)	0.22 ± 0.63	0.36 ± 1.0	0.22
Other postoperative complications			
Any complication (%)	61 (33.9)	62 (44.9)	0.045
Clavien-Dindo 1–2 complications only (%)	30 (16.7)	36 (26.1)	0.04
Clavien-Dindo 3–5 complications (%)	31 (17.2)	26 (18.8)	0.71
Comprehensive Complication Index			
Mean ± SD	8.55 ± 14.4	12.2 ± 20.1	
Length of stay			
Median (IQR) days	4 (3–6)	5 (3–8)	0.19
Over 7 days (%)	34 (18.9)	36 (26.1)	0.13
Mortality			
30 days (%)	1 (0.6)	3 (2.2)	0.2
90 days (%)	6 (3.3)	6 (4.3)	0.64

The laboratory test results of the patients are presented in Table 3.

**Table 3:** The laboratory test results of the patients

	IV-IST				Non-IV-IST		
	Pre-iron infusion	Preoperative	At discharge	One month postoperatively	Preoperative	At discharge	One month postoperatively
Hb (mean ± SD), g/l	99.2 ± 15.5	110 ± 12.5	108 ± 11.7	126 ± 12.1	113 ± 10.5	108 ± 9.6	119 ± 11.9
MCV (mean ± SD), fl	80.0 ± 8.5	83.4 ± 6.9	84.7 ± 5.8	87.6 ± 5.0	87.1 ± 6.7	87.4 ± 5.9	89.1 ± 5.8
Thrombocytes (mean ± SD) × 10^9^/l	350 ± 106	322 ± 98	317 ± 117	273 ± 91.0	301 ± 104	324 ± 154	289 ± 103
Creatinine (mean ± SD), μmol/l	76.3 ± 18.4	75.8 ± 18.8	64.5 ± 16.7	72.7 ± 16.9	79.6 ± 24.7	70.1 ± 24.3	78.0 ± 23.0
CEA (median [IQR]), μg/l		2.95 (1.70–8.98)			2.70 (1.50–6.48)		

Preoperative hemoglobin in the iv iron group was 110 ± 12.5 (mean ± SD) g/l, and in the control group, it was 113 ± 10.5 (mean ± SD) g/l.

### RBCT

No statistically significant differences were found between the groups in the number of patients receiving intraoperative or postoperative RBCT. Specifically, 15.6% (28/180) of patients in the iv iron group received intraoperative RBCT and 21.0% (29/138) in the control group (*p* = 0.21, *OR* 0.69, 95% CI 0.39–1.23) received intraoperative RBCT. Postoperatively, 11.7% (21/180) of patients in the iv iron group and 16.7% (23/138) in the control group received RBCT (*p* = 0.20, *OR* 0.66, 95% CI 0.35–1.25).

When both intraoperative and postoperative RBCTs were pooled and analyzed, a trend was found for less need (although not statistically significant) in the iv iron group: In the iv iron group, 22.8% (41/180) of the patients received either intra- or postoperative RBCT. In the control group, this was 31.9% (44/138) of the patients (*p* = 0.07, *OR* 0.63, 95% CI 0.38–1.04).

### Postoperative complications other than RBCT

Of the patients receiving iv iron, 61 out of 180 (33.9%) had at least one postoperative complication other than RBCT. In the control group, 62 out of 138 patients (44.9%) had at least one postoperative complication (*p* = 0.045, *OR* = 0.63, 95% CI 0.40–0.99). This difference was found to be explained by the difference in the number of patients representing solely mild (= a Clavien-Dindo score 1 or 2) complications. In the iv iron group, 30 out of 180 patients (16.7%) had solely mild (Clavien-Dindo 1–2) complications in contrast with the control group where 36 out of 138 patients (26.1%) were diagnosed with solely mild (Clavien-Dindo 1–2) complications (*p* = 0.04, *OR* = 0.57, 95% CI 0.33–0.98). The number of patients with severe complications (= a Clavien-Dindo score 3 or higher) did not significantly differ between the groups.

Complications with Clavien-Dindo score 1 or 2 other than RBCT are presented in Table 4.

**Table 4:** Mild (Clavien-Dindo score 1 or 2) complications other than RBCT

**Complication**	**iv iron**	**Control**
	***n* (%)**	***n* (%)**
Fever	9 (5.0)	6 (4.3)
Pneumonia	7 (3.9)	4 (2.9)
Nausea/vomiting	6 (3.3)	3 (2.2)
Hypokalemia	3 (1.7)	6 (4.3)
Atrial fibrillation	4 (2.2)	3 (2.2)
Wound infection	3 (1.7)	4 (2.9)
Urinary tract infection	3 (1.7)	3 (2.2)
Pulmonary embolism	1 (0.6)	4 (2.9)
Dyspnea	2 (1.1)	2 (1.4)
Hematochezia	1 (0.6)	2 (1.4)
Acute coronary syndrome	1 (0.6)	2 (1.4)
Wound dehiscence	1 (0.6)	1 (0.7)
Unidentified infection	0 (0)	2 (1.4)
Ascites	1 (0.6)	0 (0)
Intra-abdominal abscess	1 (0.6)	0 (0)
Pressure ulcer	1 (0.6)	0 (0)
Disorientation	1 (0.6)	0 (0)
Supraventricular tachycardia	1 (0.6)	0 (0)
Allergic reaction	1 (0.6)	0 (0)
Urinary retention	1 (0.6)	0 (0)
Cardiac insufficiency	1 (0.6)	0 (0)
Diarrhea	1 (0.6)	0 (0)
Hyponatremia	0 (0)	1 (0.7)
Intestinal obstruction	0 (0)	1 (0.7)
Pleural effusion	0 (0)	1 (0.7)
Uterine infection	0 (0)	1 (0.7)

### The prevalence of postoperative anemia

**Fig. 1:**
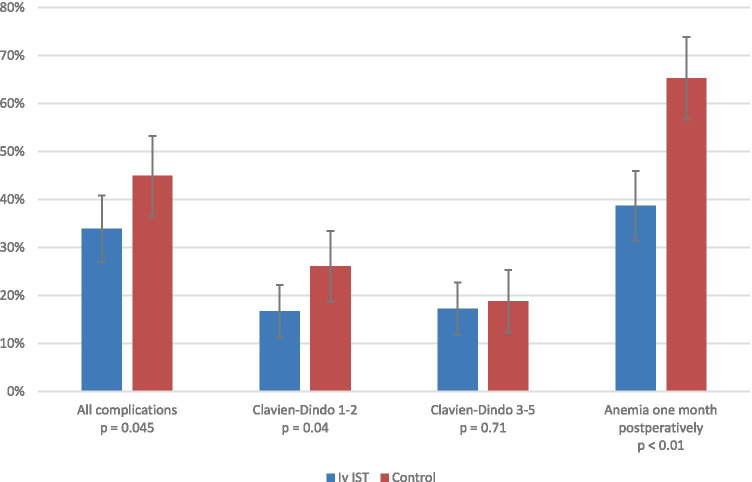
Differences between the iv iron and control groups in all complications, Clavien-Dindo 1–2 complications, Clavien-Dindo 3–5 complications, and anemia at one month after surgery. A 95% confidence interval is shown using error bars

In the iv iron group, 67 out of 173 patients (38.7%) had postoperative anemia at one month after surgery, whereas in the control group, 77 out of 118 patients (65.3%) were anemic at one month (*p* < 0.01, *OR* 0.34, 95% CI 0.21–0.55) (Fig. 1).

### Length of stay

The median LOS was 4 days for the iv iron group and 5 days for the control group. No statistically significant difference was detected between the groups (mean rank for iv iron group = 167.2 and for control group it is 153.6, *U* = 11,358, *p* = 0.19).

### Mortality

No statistically significant differences were found between the groups in 30- or 90-day mortality. In the iv iron group, 1/180 (0.6%) patients died within 30 days, whereas in the control group, 3/138 (2.2%) patients died (*p* = 0.20, *OR* 0.25, 95% CI 0.026–2.4).

In the iv iron group, 6/180 (3.3%) patients died within 90 days, whereas in the control group, 6/138 (4.3%) patients died (*p* = 0.64, *OR* 0.76, 95% CI 0.24–2.4).

## Discussion

We report no significant benefit from preoperative iv iron treatment compared to no iron in terms of RBCT-need in elective colon carcinoma patients with anemia. Previously, the opposite has been recognized in many studies observing iv iron–treated colorectal cancer patients [16, 17]. Calleja et al. reported a significantly decreased number of postoperative RBCTs and number of patients with postoperative anemia at 30 days after surgery in a iv iron group. They furthermore hypothesized that iv iron reduces the number of postoperative complications indirectly by reducing the need for RBCTs [10]. In our prehabilitated elective patient population, the perioperative hemorrhage is kept to minimum and the need for RBCTs is uniformly low. Thus, our setting does not support this sort of analysis. Further, high-quality studies are needed to corroborate the abovementioned indirect hypothesis.

In line with existing literature [9, 10], we found less postoperative anemia in the patients with preoperative iv iron compared to patients without iv iron. This indirectly suggests that our patients are mostly iron deficient preoperatively. The increased hemoglobin-level could be a significant factor in improving patient’s quality of life (QoL) postoperatively. In a study by Keeler et al. [18], postoperative QoL was compared between iv iron and oral iron groups. They reported that the iv iron group had statistically significantly better scores. Higher QoL also correlated with absolute hemoglobin levels.

This is the first study to demonstrate a significant decrease in postoperative complications in anemic colon cancer patients receiving preoperative iv iron. This is in line with our hypothesis, given the common nature of anemia in patients with colon cancer and its acknowledged role as a risk factor for surgery [3, 4]. The result is important because, until now, RBCT has been the main regimen of choice for treating perioperative anemia although its use has been associated with worse oncological outcomes [7].

In a study by Calleja et al. [10], a lower number of re-interventions and surgical complications were recorded in patients with colon cancer receiving preoperative iv iron compared with anemic control patients (20.7 vs. 26.5%), although this difference was statistically insignificant. In comparison to our study, the patients in their control group were on oral IST at the time of diagnosis, which possibly diminishes the significance of iv iron.

Furthermore, Calleja et al. reported significantly decreased postoperative LOS in their iv iron group. In contrast to their setting, all the patients in our study were treated within an updated ERAS program. As a consequence, the LOSs are optimized and short in general (with routine discharge after colon resection on the second to fourth postoperative day) and not readily truncated with further procedures (including iv iron).

This study has some limitations. Due to the retrospective nature, not all necessary iron parameters were available for all patients, thus complicating the definition of iron deficiency. This means that some patients in the iv iron group in this study were possibly not iron deficient. Due to the high prevalence of iron deficiency in colon cancer patients and the lack of other diagnoses causing microcytic anemia in the Finnish population (e.g., Thalassemia), this should not have a significant effect on the results of the study. To account for this, we did a further analysis by excluding patients without microcytosis from the iv iron group. The results were similar, and the differences between the groups were even more distinct. This could contribute to a selection bias in this study. Patients in the control group were possibly more likely to have other types of anemia (non-iron deficiency, functional iron deficiency, or iron sequestration). Patients who did not have definitive iron-deficiency anemia could have been less likely to receive preoperative iv iron and thus more likely to be included in the control group. These issues will be addressed by a prospective study, which is presently ongoing at our clinic.

## Conclusion

We report no significant reduction in RBCTs, LOS, or short-term mortality with preoperative iv-iron treatment compared to no iron in elective and preoperatively anemic patients with colon cancer. However, preoperative iv iron reduces mild (a Clavien-Dindo score 1 or 2) postoperative complications and postoperative anemia in these patients. Accordingly, systematic use of iv iron in colon cancer patients with anemia could be beneficial, but RCTs are needed on the subject.
